# Non-parametric analysis of seasonality in birth and multiple sclerosis risk in second generation of migrants in Kuwait

**DOI:** 10.1186/s12883-014-0170-7

**Published:** 2014-08-26

**Authors:** Saeed Akhtar, Raed Alroughani, Ahmad Al-Shammari, Jarrah Al-Abkal, Yasser Ayad

**Affiliations:** 1 Department of Community Medicine & Behavioural Sciences, Faculty of Medicine, University of Kuwait, Safat 13110, Kuwait; 2 Division of Neurology, Department of Medicine, Amiri Hospital, Arabian Gulf Street, Kuwait City 13041, Kuwait; 3 Neurology Clinic, Dasman Diabetes Institute, Dasman 15462, Kuwait; 4 Al-Adan Hospital, Sabah Al Salem 44403, Kuwait; 5 Department of Surgery, Farwaniya Hospital, Alrawdha 7346, Kuwait; 6 Department of Neurology, Ibn Sina Hospital, Sabah Medical Area, Safat, Kuwait

## Abstract

**Background:**

There are inconsistent reports about multiple sclerosis (MS) risk among migrants from low to high MS risk geographical regions. This study assessed the overall MS incidence and evaluated seasonality in birth and subsequent MS risk later in the life in second generation of migrants born and lived in Kuwait.

**Methods:**

We assessed the overall and gender-specific MS risk in second generation of migrants born and lived in Kuwait between January 1, 1950 and April 30, 2013. Data on migrants’ MS patients diagnosed and registered in Kuwait National MS Registry were used. Hewitt’s non-parametric test was carried out to evaluate the seasonality in migrants’ MS births in comparison with the second generation migrants’ births in general population.

**Results:**

During the study period, an overall risk of migrants’ MS births (per 100,000 non-Kuwaiti births in general population) was 23.8 (95% CI: 20.8 – 27.0). Gender-specific MS risk showed that non-Kuwaiti female had statistically significant (*p* = 0.003) higher risk (28.6; 95% CI: 24.2 – 33.7) than non-Kuwaiti males (18.7; 95% CI: 15.1–23.0). The month-specific distribution of migrants’ MS births compared with migrants’ births in general population did not differ significantly (χ^2^ goodness-of-fit test statistic = 9.51, *p* = 0.575). Hewitt’s non-parametric test revealed an evidence of slight but statistically non-significant (*p* = 0.090) increased tendency of migrants’ MS births during September through February.

**Conclusions:**

The proportion of migrants’ MS births (per 100,000 migrants’ births in general population) over the study period was 23.8 (95% CI: 20.8 – 27.0), which was statistically significantly higher than the previously reported Kuwaiti national MS births (16.2; 95% CI: 15.1–17.4) in Kuwait. Non-parametric analysis showed slight but statistically non-significant increased tendency of migrants’ MS births from September through February. Knowledge of MS risk factors and how and when they act among genetically vulnerable individuals from gestation to early adulthood will help design prevention strategies.

## Background

Multiple sclerosis (MS) is a highly debilitating immune mediated disorder of the central nervous system and is often progressive and fatal. MS etiology seems to be determined by environmental factors and genetic predisposition [1]. The contribution of each factor in MS etiology may vary in different geographic areas resulting into atypical spatial patterns of its incidence across the globe [2]. Hitherto known spatial pattern of MS reflects the highest prevalence, morbidity and mortality rates in the temperate zones of both northern and southern hemispheres and with decreasing rates in the subtropics and tropics. For instance, Europe, Canada and the northern parts of United States are high risk areas, whereas the Asia, Africa and West Indies are regarded as low risk areas [3]. So far, no specific exogenous or genetic basis for the spatial pattern has been identified, but it is speculated that some climatologic condition influences the frequency of the disease. It is unknown whether this effect is a direct on the patient or an indirect effect on the animal or plant life in his environment [1],[4].

Studies on migratory populations have indicated that the MS risk among those migrating from a high to a low risk area exceeds that of the population into which they have immigrated. Furthermore, those who migrate from a high-risk area to a low-risk area keep their high risk unless they migrate below the age of sixteen [5]-[7]. There are inconsistent reports regarding MS risk among immigrants from low risk areas such as Asia, East Africa and Caribbean to high MS risk areas. Additionally, as noted earlier, MS risk depends on age at the time of migration [8]. Reportedly, MS was relatively uncommon among West Indian and Asian immigrants to UK [8]-[10]. However, children of Asian and West Indian immigrants born in England and Wales had MS prevalence similar to that in the general population of England and Wales [11],[12].

Based on Kurtzke classification, Arabian Gulf region is located in a low-risk zone for MS [13]. However, recent studies suggest a moderate-to-high MS prevalence (range: 31–55 cases × 10^−5^ individuals) with an increase in incidence in recent years [14],[15]. Arabian Gulf countries including Kuwait have a large population of migrant workers from Asian and African countries. Prior to first Gulf War in 1990, MS prevalence in Kuwait was significantly higher among non-Kuwaiti (mainly Palestinians) (23.8 × 10^−5^) than Kuwaiti (9.5 ×10^−5^) [16]. However, the composition of population in Kuwait radically changed due to sociopolitical reasons since the first Gulf War in 1990. There was an influx of migrant workers from South and South-east Asia and other Arab countries replacing a pre-war a major expatriate group of Palestinians [15]. Furthermore, during the post- Gulf War era, the MS prevalence in Kuwait increased from 6.7 × 10^−5^ in 1993 to 14.8 ×10^−5^ in 2000. Moreover, in a complete reversal of the pattern observed before 1990, this difference was more pronounced among Kuwaiti (31.2 × 10^−5^) than non-Kuwaiti (5.6 × 10^−5^) migrant workers. Therefore, in a geographic area with previously low MS prevalence, the local environment may be responsible for the dramatic change in MS risk [15]. Seasonal pattern in MS births has been consistent of several studies conducted in a number of regions and countries [17]. Month of birth is a surrogate for continuous environmental trait which might be present during the entire year and peak at certain period rather than being present only on month or season [1]. Previously in Kuwait, we demonstrated birth of month effect on the MS risk among the Kuwaiti born in Kuwait using parametric statistical models. However, similar relationship did not hold for non-Kuwaiti born in Kuwait [18]. This study assessed the overall MS incidence and evaluated the month of birth effect on MS risk among non-nationals born, lived and diagnosed with MS in Kuwait from January 1950 to April 2013 using non-parametric analysis of the data from Kuwait National MS Registry.

## Methods

### Study setting

Kuwait is a small oil-rich country with a total approximate area of 18,000 km^2^ and a total population of 3.5 million. Kuwait is located at north-west corner of the Arabian Gulf between latitudes 28° 45′ N and 30° 05′ N and longitudes 46° 30′ and 48° 30′. The state is bounded by Saudi Arabia on the south and by Iraq on the north and west. The weather is typical of the Sahara geographical region: winter is short and cool with a mean maximum temperature of 17.7°C and a mean minimum temperature of 7.7°C in January; summer is long and hot with a mean maximum temperature of 46.6°C in July; spring and fall are short. Kuwait has a mean annual rainfall of 79.1 mm and a mean daily sunshine of 8:39 h [16]. Most of the population inhabits the eastern part of the country, which is close to the Arabian Gulf. It is estimated that only 8% of the Kuwait land area is inhabited [19]. Non-nationals constitute about 68% of the population and resultantly 80% of the labour force comprises migrants. The majority of migrants are male with a low educational attainment [20]. Amongst nationals the male and female literacy rates (defined as individuals aged 10 years or more and who can read and write) are 98.5% and 91.2%, respectively, with a gender ratio (male/female) of 1:1.04 at birth [15]. Petroleum and petrochemical products constitute the major industry [16].

### Data

A detailed description of Kuwait MS registry has been provided elsewhere [14],[18]. Briefly, a Kuwait National MS Registry was established in October 2010 after combining the database of all major hospitals including MS clinics which together accounted for nearly 98% of the MS patients diagnosed in Kuwait. Patients who met the revised 2010 McDonald Criteria for the MS diagnosis were included in the registry [21]. Prior to the application of the revised 2010 McDonald criteria, MS diagnosis was based on the previously accepted diagnostic criteria such as Schumacher, Poser or earlier versions of McDonald criteria [22]-[24]. Given the stringent definitions, MS patients diagnosed according to the older diagnostic criteria, would have also satisfied the 2010 revised version of McDonald criteria and hence included in the MS registry. Data on demographics (date of birth, gender and nationality) and clinical characteristics were extracted from patients’ medical records maintained at respective hospitals for inclusion in the registry. The reference population data for monthly births’ by gender and nationality were obtained from the records of Public Authority for Civil lnformation, Ministry of Interior, Kuwait.

### Statistical analysis

All the non-Kuwaiti MS patients diagnosed and registered between January 1, 1950 and April 30, 2013 in Kuwait National MS Registry were included. The reference group included all the non-Kuwaiti births in general population during the interval matched with that of non-Kuwaiti MS patients. The overall proportion (95% confidence interval (CI) of MS births of total non-Kuwaiti births in population was computed. Proportions (95% CI) of MS births by gender were also computed using the total non-Kuwaiti births in population. Hewitt’s non-parametric test was used to evaluate the seasonality in non-nationals’ MS births in Kuwait and subsequent risk of MS later in the life.

### Ethical considerations

We used the anonymized registry data, therefore, ethical approval of the study was neither required nor was it solicited.

## Results

During the study period, non-nationals born in Kuwait had an overall risk of MS births (per 10^5^ non-Kuwaiti births in general population) as 23.8 (95% CI: 20.8 – 27.0). Gender-specific risk of MS births (per 10^5^ non-Kuwaiti births by gender in general population) showed non-Kuwaiti female had statistically significant (*p* = 0.003) increased risk (28.6; 95% CI: 24.2 – 33.7) than non-Kuwaiti males (18.7; 95% CI: 15.1–23.0) (Table 1). Non-Kuwaiti females born and lived in Kuwait were 1.5 times more likely to have been diagnosed with MS than non-Kuwait males born and lived in Kuwait (Relative risk =1.5; 95% CI: 1.2 – 2.0). The month-specific distribution of non-Kuwaiti MS births compared with non-Kuwaiti births in general population did not differ significantly (*χ*^2^ goodness-of-fit test statistic = 9.51, *p* = 0.575). Of rank-sums for successive 6-month segments based on the Hewitt’s non-parametric test, from September through-February period showed an evidence of slight but statistically non-significant (*p* = 0.090) increased tendency of non-Kuwaiti MS births (Tables 2 and 3).

**Table 1:** Monthly distribution of multiple sclerosis births among non-nationals in Kuwait: January 1, 1950 – April 30 2013

**Month**	**Multiple sclerosis births January 1, 1950 –April 30, 2013**		**Total number of births among non-nationals**
	**Observed MS births**	**Expected MS births**	
Jan	18	21	86407
Feb	22	18	74709
Mar	17	19	81832
Apr	28	19	80336
May	15	20	82850
June	14	19	80437
July	17	20	84314
Aug	20	20	83482
Sept	20	20	83319
Oct	24	21	86844
Nov	22	20	84502
Dec	20	21	88000

**Table 2:** Month-specific total number of non-national births, multiple sclerosis births, estimates of expected relative incidence, observed relative incidence (ORI) and ORI ranks: Kuwait, January 1, 1950 – April 30, 2013

**Months**	**Non-national births (n = 997269)**	**MS births (n = 237)**	**Expected relative incidence (×10**^**3**^**)**	**Observed relative incidence (×10**^**3**^**)**	**ORI rank**
Jan	86425	18	0.020184	0.017689	5
Feb	74731	22	0.018231	0.022583	10
Mar	81849	17	0.020184	0.01764	4
Apr	80364	28	0.019533	0.028637	12
May	82865	15	0.020184	0.015374	2
June	80451	14	0.019533	0.014303	1
July	84331	17	0.020184	0.017121	3
Aug	83502	20	0.020184	0.020342	8
Sept	83339	20	0.019533	0.019725	7
Oct	86868	24	0.020184	0.023465	11
Nov	84524	22	0.019533	0.021393	9
Dec	88020	20	0.020184	0.019298	6

**Table 3:** Hewitt’s non-parametric analysis of MS births among non-nationals in Kuwait: January 1, 1950 – April 30, 2007

**Hewitt’s test for six month segments**	**Rank-sum**	***P-value***
Jan-Jun	34	> 0.25
Feb-Jul	32	> 0.25
Mar-Aug	30	> 0.25
Apr-Sep	33	> 0.25
May-Oct	32	> 0.25
Jun-Nov	39	> 0.25
Jul-Dec	44	0.242
Aug-Jan	46	0.155
Sep-Feb	48	0.090
Oct-Mar	45	0.197
Nov-Apr	46	0.155
Dec-May	39	> 0.25

## Discussion

In this evaluation, the proportion of migrants MS births over the study period was statistically significantly higher than previously reported Kuwaiti MS births [18]. Non-parametric analysis revealed slight but statistically non-significant increased tendency of migrants’ MS births from September through February. There are inconsistent reports on the question of MS risk in migrant populations. The MS risk among migrants seems to be dependent not only on a sufficiency of people who change their residence from one risk area to another but also on ages at immigration and their length of stay (exposure period) in the new land. Additionally, this relationship of geography and MS risk is further confounded by predilection for MS regardless of region [25],[26]. There is evidence from mortality data that moving from low-risk to high-risk MS regions does increase the risk of dying from MS [27]. Unpublished data also showed an increased MS risk among southern-born US-veterans who moved to North of US and Vietnamese who moved to France, though these results were considered non-confirmatory [27]. UK born-children of West Indian immigrants had the same magnitude of probable MS incidence and prevalence as was among Irish population in Northern Ireland. This finding indicated that although genetic factors play role but there is strong evidence that the cause of disease is mainly environmental and therefore preventable [28].

In line with earlier studies investigating the genetic and spatial epidemiology of this complex disease, more recent studies have highlighted how MS arises from a combination of genetic susceptibility and environmental exposures acting from gestation to early adulthood. For example, in a recent meta-analysis it has been argued that latitude-dependent month of birth effect on MS risk is likely due to ultraviolet light exposure and maternal vitamin D level [29]. Conversely, Fiddes et al. reasoned-out that confounding underlies this apparent association of month of birth and MS risk. The authors claimed that using national birth statistics as controls may generate false-positive association of MS with month of birth [30]. However, a limitation of aforementioned study is that the researchers did not analyze the original studies that highlighted the month of birth effect in MS [30]. Instead, they performed a separate study on national un-weighted births statistics, thereby showing potential sources of bias in such studies [30],[31]. Nevertheless, in search of causal relationships, season of birth, maternal Vitamin D deficiency, Epstein Barr virus infection, and smoking behaviour are strongly implicated and seem to influence genetic predisposition to MS. Additionally, these factors appear to act synergistically and the risk of MS in individuals exposed to more than one factor combines multiplicatively [1],[4],[31],[32]. Further research is needed to fully understand the environmental risk factors for MS and in this regard longitudinal studies of at-risk populations should be considered [32]. Knowledge of such risk factors may help design preventive strategies to minimize MS risk.

## Conclusions

The proportion of migrants’ MS births (per 10^5^ non-national births in general population) over the study period was 23.8 (95% CI: 20.8 – 27.0), which was statistically significantly higher than previously reported Kuwaiti MS births (16.2; 95% CI: 15.1 – 17.4) in Kuwait. Non-parametric analysis revealed slight but statistically non-significant increased tendency of migrants’ MS births from September through February. Knowledge of MS risk factors and how and when they act among genetically vulnerable individuals from gestation to early adulthood will help design prevention strategies.

## Competing interests

The authors declare that they have no competing interests.

## Authors’ contributions

SA conceived the research idea and design of the study, analyzed and interpreted the data, drafted the manuscript, critically revised the manuscript, RA helped in setting up Kuwait Multiple Sclerosis Registry, provided administrative and technical support, supervised and facilitated data acquisition, interpreted the results, critically reviewed the draft. AA, JA, YA helped in data acquisition and reviewed the draft. All authors read and approved the final manuscript.
